# Secretion of small/microRNAs including miR-638 into extracellular spaces by sphingomyelin phosphodiesterase 3

**DOI:** 10.3892/or.2014.3605

**Published:** 2014-11-13

**Authors:** SHIORI KUBOTA, MITSURU CHIBA, MIKI WATANABE, MAKI SAKAMOTO, NARUMI WATANABE

**Affiliations:** 1 Department of Medical Technology, Hirosaki University School of Health Sciences, Hirosaki, Aomori 036-8564, Japan; 2 Department of Biomedical Sciences, Division of Medical Life Sciences, Hirosaki University Graduate School of Health Sciences, Hirosaki, Aomori 036-8564, Japan; 3 Research Center or Biomedical Sciences, Hirosaki University Graduate School of Health Sciences, Hirosaki, Aomori 036-8564, Japan

**Keywords:** microRNA, miR-638, extracellular RNA, sphingomyelin phosphodiesterase 3, neutral sphingomyelinase 2, GW4869, exosome, extracellular vesicle

## Abstract

A recent study demonstrated that intracellular small/microRNAs are released from cells, and some of these extracellular RNAs are embedded in vesicles, such as ceramide-rich exosomes, on lipid-bilayer membranes. In the present study, we examined the effects of sphingomyelin phosphodiesterase 3 (SMPD3), which generates ceramide from sphingomyelin, on the release of small/microRNAs from intracellular to extracellular spaces. In these experiments, SW480 human colorectal and HuH-7 human hepatocellular cancer cells were cultured for 48 h in serum-free media. Culture supernatants were then collected, and floating cells and debris were removed by centrifugation and filtration through a 0.22-μm filter. Extracellular small RNAs in purified culture supernatants were stable for 4 weeks at room temperature, after 20 freeze-thaw cycles and exposure to pH 2.0, and were resistant to ribonuclease A degradation. Amino acid sequence analyses of SMPD3 showed high homology between mammals, indicating evolutionary conservation. Therefore, to investigate the mechanisms of cellular small/microRNA export, SW480 and HuH-7 cells were treated with the SMPD3 inhibitor GW4869 in serum-free media. Culture supernatants were collected for microarray and/or reverse transcription quantitative polymerase chain reaction (RT-qPCR) experiments. The number of microRNAs in culture supernatants was decreased following treatment with GW4869. Among these, extracellular and intracellular miR-638 were dose-dependently decreased and increased, respectively. These data suggest that SMPD3 plays an important role in the release of microRNAs into extracellular spaces.

## Introduction

MicroRNAs of 20–25 nucleotides act as post-transcriptional regulators of gene expression, and their localization suggests that they primarily function in the cytoplasm. Recently, a number of microRNAs were found in extracellular spaces (1), and some of these were embedded in extracellular vesicles such as exosomes (2). However, it has been suggested that some extracelluar microRNAs form complexes with Argonaute 2 (Ago2), high-density lipoprotein (HDL) and other RNA-binding proteins (3–6). Therefore, microRNAs may be present in various bound forms in extracellular spaces. Diagnostic biomarkers were recently identified in body fluids such as serum, plasma, urine, milk and saliva (7–11). Among these biomarkers, some extracellular RNAs were shown to be uniquely stable in the presence of ribonuclease (12–14). However, these data require further validation in focused studies of extracellular microRNA stability.

Exosomes are extracellular vesicles, ~40–200 nm in diameter, which are secreted from epithelial (15), endothelial (16), cancer (17), dendritic (18), and mesenchymal stem cells (19), as well as B lymphocytes (20). Exosome secretion has been identified in human and mouse cells *in vitro* (1). However, few studies have demonstrated RNA secretion in other organisms.

Although the mechanisms of exosome biogenesis remain to be adequately defined, current models suggest that exosomes are formed within multivesicular bodies (MVBs) (21), which are formed during maturation of early into late endosomes, with concomitant and corresponding accumulation of intraluminal vesicles (ILVs) (22). Endosomal sorting complexes required for transport (ESCRT) machinery are also responsible for generating vesicles in MVBs through a process known as endosome budding (23). In addition, ceramide is reportedly involved in an ESCRT-independent process of exosome generation (24). Ceramide, which is generated from sphingomyelin by neutral sphingomyelinase 2 (nSMase2), is found in lipid components of exosome membranes (25), and is encoded by the sphingomyelin phosphodiesterase 3 (*SMPD3*) gene. Although this enzyme has been shown to be involved in the secretion of small RNAs such as microRNAs (26), which small/microRNAs are released following the actions of nSMase2 remains to be determined.

In the present study, we investigated the stability of extracellular small RNAs against external factors including ribonuclease A (RNase A), long-term incubation, freeze-thaw, and pH change using HuH-7 human hepatocellular cancer cells. In addition, we examined the evolutionary conservation of SMPD3 in mammals and determined the effects of an SMPD3 inhibitor on the release of small/microRNAs from HuH-7 and SW480 human colorectal cancer cells.

## Materials and methods

### Cell lines and culture

HuH-7 human hepatocellular cancer cells (JCRB0403) were purchased from the Health Science Research Resources Bank (Osaka, Japan). The human colorectal cancer cell line SW480 (CCL-228) was purchased from the American Type Culture Collection (ATCC, Manassas, VA, USA). HuH-7 cells were cultured in Dulbecco’s minimal essential medium (D-MEM; Wako, Tokyo, Japan) supplemented with 10% fetal bovine serum (FBS; Life Technologies, Carlsbad, CA, USA), 100 U/ml penicillin, and 100 μg/ml streptomycin. SW480 cells were cultured in RPMI-1640 medium (Wako) supplemented with 10% FBS, 100 U/ml penicillin, and 100 μg/ml streptomycin. The cells were cultured at 37°C in a 5% CO_2_ atmosphere.

### Purification of culture supernatants

SW480 and HuH-7 cells were plated on collagen-coated 10-cm dishes at 1×10^6^ cells/dish in culture media. After 72 h, the culture media were discarded and the cells were washed three times in serum-free culture media. Serum-free culture media supplemented with the SMPD3 inhibitor GW4869 (Sigma-Aldrich, St. Louis, MO, USA) at final concentrations of 0, 1.0, 3.3, and 10.0 μM were then added at 10 ml per dish, and the cells were cultured for 48 h. Cell culture media were then collected and centrifuged at 300 × g at 4°C for 3 min to remove floating cells. Supernatants were centrifuged at 2,000 × g at 4°C for 15 min and were collected in new tubes. Culture supernatants were also centrifuged at 12,000 × g at 4°C for 35 min to remove cell debris, and the supernatants were collected in new tubes and filtered using 0.22-μm filters. Extracellular RNAs in the supernatants were then isolated using Isogen II (NipponGene, Tokyo, Japan).

### RNA extraction

Extracellular and intracellular RNAs from SW480 or HuH-7 cells were isolated using Isogen II (NipponGene) according to the manufacturer’s instructions. The sizes of extracted RNAs were determined using an Agilent 2100 Bioanalyzer and Agilent RNA 6000 Pico kits (both from Agilent Technologies, Foster City, CA, USA) according to the manufacturer’s instructions.

### Microarray analysis

Species of extracellular microRNAs were distinguished by labeling with Hy5 fluorescent dye using a miRCury LNA™ microRNA Hy5 Power labeling kit (Exiqon, Copenhagen, Denmark). Microarray analyses were then conducted using a Toray microRNA microarray system. Toray 3D-Gene human miRNA oligo chips (Toray, Tokyo, Japan) were hybridized with Hy5-labeled microRNAs in hybridization solution at 32°C for 16 h using a hybridization oven. Hybridized microarray chips were then washed in a wash buffer according to the manufacturer’s instructions, and images of fluorescent signals were captured using a Toray 3D-gene scanner 3000 (Toray).

### Reverse transcription polymerase chain reaction (RT-PCR) and RT quantitative PCR (RT-qPCR)

To investigate *SMPD3* mRNA expression in SW480 and HuH-7 cells, cDNAs were synthesized from isolated RNAs using High Capacity cDNA reverse transcriptase kits according to the manufacturer’s instructions. Subsequently, qPCR for mRNAs was performed using 2X Power SYBR-Green master mix, 10.0 μM forward and reverse primers (Table I), and a StepOne Plus real-time PCR system (all from Life Technologies), under the following conditions: 10 min at 95°C, followed by 40 cycles at 95°C for 15 sec and 60°C for 60 sec. GAPDH was used as an internal control. Expression levels were determined using the comparative Ct method and were normalized to those from SW480 cells. Amplified fragments were then detected on 4% agarose gel electrophoresis containing ethidium bromide using a ChemiDoc XRS system and Quantity One software (both from Bio-Rad, Hercules, CA, USA).

Expression levels of extracellular and intracellular microRNAs from SW480 and HuH-7 cells were analyzed using cDNAs that were synthesized from microRNAs using TaqMan microRNA RT kits and the prescribed 5X RT primer (both from Life Technologies) according to the manufacturer’s instructions. Subsequently, qPCR for microRNAs was performed using FastStart TaqMan probe master (Roche Diagnostics, Basel, Switzerland), a 20X probe, and a StepOne Plus real-time PCR system (Life Technologies) under the following conditions: 10 min at 95°C, followed by 40 cycles at 95°C for 15 sec and 60°C for 60 sec. RNAs were isolated from 200-μl aliquots of culture supernatants following the addition of 1 μl of 5 nM cel-miR-39. Cel-miR-39 was used as an external control and U6 small nuclear RNA (snRNA) was used as an internal control. Expression levels were determined using the comparative Ct method.

### Multiple alignments of SMPD3 amino acid sequences

Amino acid sequences for *Homo sapiens* SMPD3, NP_061137.1; *Pan troglodytes* SMPD3, XP_001167790.1; *Mus musculus* SMPD3, NP_067466.1; and *Bos taurus* SMPD3, NP_001179292.1, were obtained from the NCBI database (http://www.ncbi.nlm.nih.gov), and were subjected to multiple alignment analysis using Genetyx 10 software (Genetyx, Tokyo, Japan).

### Statistical analysis

Data are presented as the mean ± standard error of the mean (SEM). Multiple group comparisons were performed using one-way analysis of variance (ANOVA), followed by post hoc pair-wise comparisons of significant differences using Dunnett’s test. Differences were considered significant when P<0.01.

## Results and Discussion

### Extracellular small RNAs are stable against changes in various conditions

Encapsulation of released cellular small RNAs in exosomes likely allows high stability against changes in several conditions (12–14). Accordingly, small RNAs in purified supernatants from HuH-7 cells were stable through RNase A treatment, long-term incubation, cycles of freezing and thawing and pH changes.

**Figure 1:**
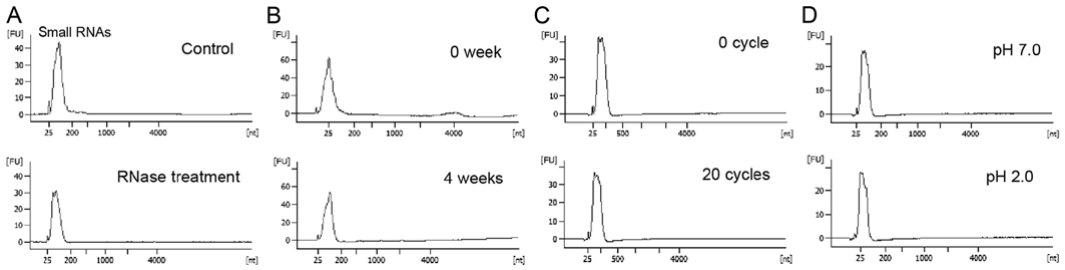
Extracellular small RNAs in cell culture media are stable against several external conditions. (A–D) Stability of extracellular small RNAs from HuH-7 cells, which were seeded at 1×10^5^ cells/well in 12-well plates. After 24 h, cells were washed three times in serum-free media. Serum-free (1 ml) media were then added, and the cells were incubated at 37°C for 48 h. Culture supernatants were then collected and purified by centrifugation and filtration. Culture supernatants of HuH-7 cells were (A) treated with a final concentration of 4 μg/ml of ribonuclease A (RNase A) at 37°C for 30 min, (B) incubated for 4 weeks at room temperature, (C) subjected to 20 freeze-thaw cycles, and (D) were subjected to a pH decrease to 2.0. Small RNAs were extracted from 200 μl aliquots and were detected using an Agilent bioanalyzer.

In experiments conducted in this study, serum-free culture supernatants from HuH-7 cells were purified by centrifugation and filtration and were incubated with RNase A at a final concentration of 0.4 μg/ml for 10 min at 37°C. After extraction of total RNAs from culture supernatants, a peak for small RNAs of 25–200 nt was detected using an Agilent bioanalyzer (Fig. 1A). However, in culture supernatants, small RNAs were stable after incubation at room temperature for 4 weeks, 20 cycles of freezing and thawing (room temperature to −80°C), and reduction of pH to 2.0 (Fig. 1B–D). These data indicate high stability of small RNAs in culture supernatants.

### Evolutionary conservation of SMPD3 in mammals

**Figure 2:**
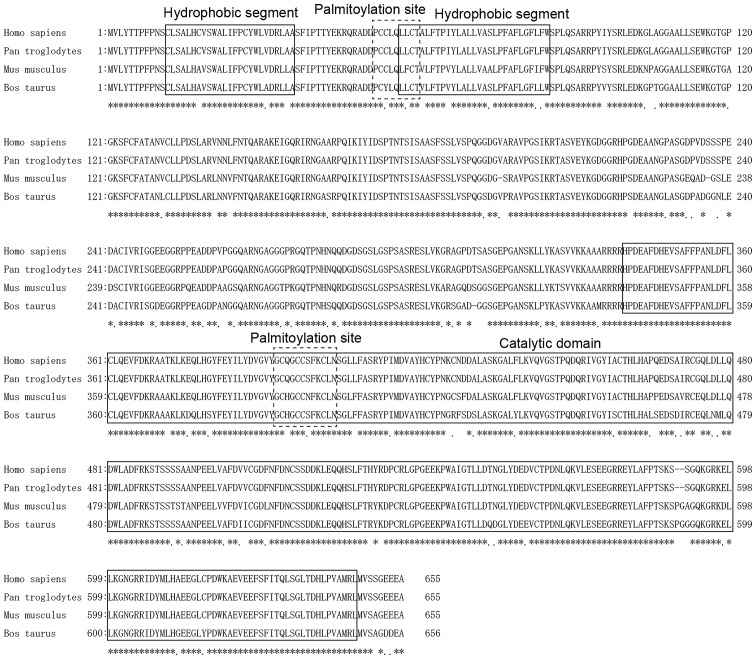
Evolutionary conservation of sphingomyelin phosphodiesterase 3 (SMPD3) in mammals. Amino acid sequence alignment analyses of SMPD3; two hydrophobic segments, two palmitoylation sites, and a catalytic domain were highly conserved in *Homo sapiens*, *Pan troglodytes*, *Mus musculus*, and *Bos taurus*. Asterisks indicate matched amino acids in all 4 sequences, while dots indicate matched amino acids in 3 of 4 mammals.

SMPD3 is reportedly involved in the secretion of microRNAs (26). The present analyses of various mammalian SMPD3 sequences (*Homo sapiens*, *Pan troglodytes*, *Mus musculus* and *Bos taurus*) indicated high sequence homology (Fig. 2), with an amino acid sequence identity of 99.69, 91.02 and 89.50% between *Homo sapiens* and *Pan troglodytes*, *Mus musculus*, *Bos Taurus*, respectively. Moreover, two hydrophobic segments, two palmitoylation sites and the catalytic domain were highly conserved between examined mammals (Fig. 2). These analyses suggest that SMPD3 may have similar functions across these species.

### Small/microRNAs, such as miR-638, are secreted into extracellular spaces via a ceramide-dependent mechanism

**Figure 3:**
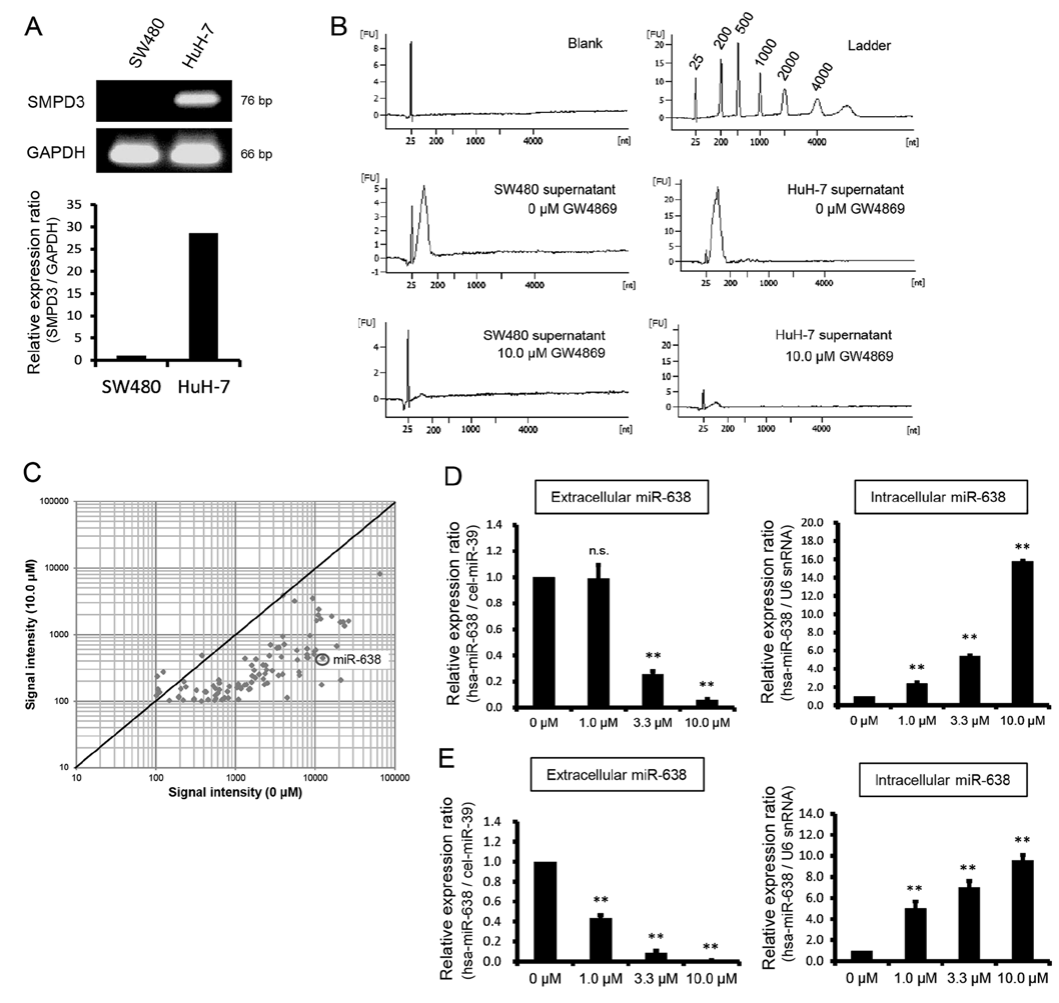
SMPD3 is involved in the secretion of small/microRNAs from cells. (A) Comparison of SMPD3 mRNA expression in SW480 and HuH-7 cells by electrophoresis of reverse transcription polymerase chain reaction (RT-PCR) products and RT quantitative PCR (RT-qPCR) using the primer pairs described in Table I. GAPDH was used as an internal control. Expression levels were determined using the comparative Ct method and were normalized to those of SW480 cells. (B) Effects on the quantities of small RNAs released from SW480 or HuH-7 cells after treatment with the SMPD3 inhibitor GW4869. SW480 and HuH-7 cells were seeded at 1×10^5^ cells/well in 12-well plates. After 24 h, the cells were washed three times in serum-free media and were incubated in 1 ml of serum-free media at 37°C for 48 h. Culture supernatants were collected and purified by centrifugation and filtration and extracellular RNAs were isolated from 400-μl aliquots. Quantitative analysis of small RNAs in culture supernatants of SW480 or HuH-7 cells treated with GW4869 using an Agilent bioanalyzer. The quantities of small RNAs in culture supernatants decreased following treatment with 10.0 μM GW4869. (C) Scatter plot of microRNA expression in culture supernatants from HuH-7 cells after treatment with 0 and 10.0 μM GW4869. Analyses were performed using a Toray microRNA microarray system. (D and E) Expression levels of extracellular and intracellular miR-638 in SW480 and HuH-7 cells treated with GW4869. Samples of cell pellets and supernatants from (D) HuH-7 cultures (each n=8) and (E) SW480 (each n=6) were prepared, RNA was extracted, and RT-qPCR analyses were performed. Cel-miR-39 and U6 small nuclear RNA (snRNA) were used as internal controls. Bars indicate the mean ± standard error of the mean (SEM) values. Multiple comparisons were made using one-way ANOVA followed by Dunnett’s multiple comparisons post hoc test. Double asterisks indicate significant differences (P<0.01 vs. 0 μM); n.s., not significant.

Although nSMase2 produces ceramide from sphingomyelin (25), it is reportedly involved in the secretion of small RNAs such as microRNAs (26). Thus, we investigated the relationship between small RNA secretion and *SMPD3* mRNA expression in SW480 and HuH-7 cells. In the RT-qPCR experiments, *SMPD3* mRNA expression in HuH-7 cells was 28.62-fold higher than that in SW480 cells (Fig. 3A). Moreover, peak RNA release from HuH-7 cells was higher than that of SW480 cells (Fig. 3B), and corresponded with high *SMPD3* mRNA expression.

In the present study, concentrations of small RNAs in culture supernatants were determined following treatment of HuH-7 or SW480 cells with non-competitive SMPD3 inhibitor GW4869 at a final concentration of 10.0 μM. In these experiments, small RNA contents were markedly decreased after 48 h (Fig. 3B), suggesting that SMPD3 is important in small RNA secretion.

In subsequent experiments, the amounts and species of microRNAs in culture supernatants from GW4869-treated HuH-7 cells were analyzed using a Toray microRNA microarray system. MicroRNA expression profiles following treatment with 0 and 10.0 μM GW4869 for 48 h are shown in a scatter plot (Fig. 3C). Various microRNAs, including miR-638, were decreased in culture supernatants from GW4869-treated cells (Table II).

Subsequent RT-qPCR experiments showed a significant decrease in miR-638 expression in the SW480 and HuH-7 cells and supernatants following treatment with GW4869 (P<0.01). Specifically, extracellular miR-638 expression in HuH-7 cells was decreased 3.92- and 16.67-fold in the presence of 3.3 and 10.0 μM GW4869, respectively. In SW480 cells it was decreased 2.29-, 11.36- and 113.14-fold in the presence of 1.0, 3.3 and 10.0 μM GW4869, respectively (Fig. 3D–E). By contrast, intracellular miR-638 expression was significantly increased 2.43-, 5.41- and 15.81-fold in the presence of 1.0, 3.3, and 10.0 μM GW4869 in HuH-7 cells, and 5.01-, 7.00-, and 9.57-fold, respectively, in SW480 cells when compared with expression in the presence of 0 μM GW4869 (P<0.01; Fig. 3D–E). These data suggest that SMPD3 plays an important role in the release of a number of microRNAs, including miR-638, and that microRNAs accumulated in cells following the inhibition of exosome membrane formation by GW4869.

Small RNAs secretions are involved in the formation of exosomes, the regulation of vesicle trafficking, and the plasma membrane fusion of MVBs (28,29). Exosome transport is considered a highly controlled process that involves a number of Rab GTPases. Accordingly, Rab11 overexpression has been shown to stimulate exocytosis (30), and Rab27a and Rab27b have been shown to control exosome secretion by regulating vesicle transport of MVBs to plasma membranes (31). In addition, secretion of exosomes containing small RNAs requires fusion of MVBs to plasma membranes, potentially involving soluble N-ethylmaleimide-sensitive factor attachment protein receptor (SNARE) protein complexes (32). Future studies are required to clarify the mechanisms of RNA and exosome release into extracellular spaces.

In conclusion, extracellular small RNAs are comparatively stable due to their presence in exosomes. Moreover, the high evolutionary conservation of SMPD3 indicates an important role in the release of miR-638 and other microRNAs into extracellular spaces.

## Figures and Tables

**Table I:** Primer sequences for RT-qPCR.

Gene name	Primer sequence	Size (nt)	Amplicon size (bp)
SMPD3	F: 5′-CGTCGTCTGTGGAGATTTCA-3′	20	76
SMPD3	R: 5′-GGTGAACAGGGAGTGTTGCT-3′	20	
GAPDH	F: 5′-AGCCACATCGCTCAGACAC-3′	19	66
GAPDH	R: 5′-GCCCAATACGACCAAATCC-3′	19	

**Table II:** The microRNA expression in culture supernatants of HuH-7 cells treated with 0 or 10.0 μM GW4869 using a Toray microRNA microarray system.

	Raw signal intensity	
	
microRNAs	0 μM GW4869	10.0 μM GW4869
hsa-miR-3960	65,098.0	8,112.9
hsa-miR-4787-5p	26,349.0	1,593.3
hsa-miR-4508	23,348.0	1,349.6
hsa-miR-3665	22,420.0	1,538.6
hsa-miR-4484	21,004.0	207.2
hsa-miR-762	20,776.0	1,527.1
hsa-miR-4739	18,737.0	671.5
hsa-miR-4516	16,181.0	1,884.0
hsa-miR-4505	12,498.5	443.2
hsa-miR-3648	12,080.7	175.5
hsa-miR-4466	11,807.8	1,714.7
hsa-miR-4488	11,136.4	2,385.7
hsa-miR-3196	11,008.8	1,971.5
hsa-miR-2861	10,473.1	1,617.8
hsa-miR-638	10,180.6	587.0
hsa-miR-1908	9,862.2	559.4
hsa-miR-4725-3p	9,736.2	502.2
hsa-miR-4294	9,309.6	3,504.9
hsa-miR-3656	8,587.1	964.6
hsa-miR-4467	8,139.1	446.2
hsa-miR-4745-5p	7,940.2	495.4
hsa-miR-4734	7,933.7	615.8
hsa-miR-4497	7,932.5	451.3
hsa-miR-744	6,356.6	244.6
hsa-miR-4327	6,287.8	272.2
hsa-miR-4723-5p	6,007.8	416.2
hsa-miR-663	5,899.5	576.6
hsa-miR-3621	5,509.6	3,186.3
hsa-miR-4454	4,467.7	112.9
hsa-miR-1268	4,115.5	711.4
hsa-miR-1246	3,965.1	3,866.2
hsa-miR-3940-5p	3,925.4	936.0
hsa-miR-4492	3,916.0	251.9
hsa-miR-3178	3,777.1	459.8
hsa-miR-1469	3,516.2	639.1
hsa-miR-4530	3,463.2	242.0
hsa-miR-1228^*^	3,319.6	628.7
hsa-miR-4459	2,894.8	286.1
hsa-miR-4687-3p	2,760.3	700.9
hsa-miR-4463	2,668.5	478.1
hsa-miR-1915	2,516.5	185.4
hsa-miR-1260b	2,456.4	295.3
hsa-miR-4281	2,407.2	344.2
hsa-miR-149^*^	2,356.0	257.3
hsa-miR-4749-5p	2,305.3	176.4
hsa-miR-1275	2,241.6	215.3
hsa-miR-1268b	2,210.7	350.2
hsa-miR-3141	1,979.9	250.6
hsa-miR-4417	1,809.8	425.3
hsa-miR-4763-3p	1,774.2	223.5
hsa-miR-4651	1,719.9	290.3
hsa-miR-4442	1,629.6	238.6
hsa-miR-4741	1,607.0	345.4
hsa-miR-3197	1,600.5	192.0
hsa-miR-3937	1,528.1	152.4
hsa-miR-4532	1,467.0	149.0
hsa-miR-4655-5p	1,377.6	177.1
hsa-miR-3180	1,312.2	435.1
hsa-miR-4695-5p	1,298.0	174.7
hsa-miR-3180-3p	1,172.2	153.8
hsa-miR-92b^*^	1,163.7	158.8
hsa-miR-671-5p	1,091.4	106.2
hsa-miR-1280	1,085.7	180.6
hsa-miR-4728-5p	1,051.4	159.1
hsa-miR-4689	836.7	166.4
hsa-miR-642b	808.9	111.5
hsa-miR-1909	791.7	139.2
hsa-miR-939	730.0	112.2
hsa-miR-4656	677.5	105.1
hsa-miR-3195	631.0	186.6
hsa-miR-3663-3p	630.8	147.8
hsa-miR-92a-2^*^	621.2	207.6
hsa-miR-3188	606.1	132.1
hsa-miR-4486	565.7	109.2
hsa-miR-1202	559.6	154.5
hsa-miR-4270	557.6	121.8
hsa-miR-3185	547.7	102.1
hsa-miR-4649-5p	526.5	169.7
hsa-miR-4688	454.0	105.3
hsa-miR-4707-5p	428.8	136.7
hsa-miR-4697-5p	414.7	138.1
hsa-miR-4732-5p	381.2	481.5
hsa-miR-4758-5p	374.8	100.0
hsa-miR-720	323.1	145.2
hsa-miR-296-5p	301.6	108.3
hsa-miR-4429	300.4	105.1
hsa-miR-4433	238.7	163.4
hsa-miR-1290	226.1	196.4
hsa-miR-483-3p	221.0	103.3
hsa-miR-4730	206.3	152.8
hsa-miR-542-5p	193.6	113.9
hsa-miR-4787-3p	193.4	116.6
hsa-miR-320b	148.3	102.1
hsa-miR-4443	123.7	272.6
hsa-miR-22	111.2	135.5
hsa-miR-21	105.8	149.9
hsa-miR-122	104.1	234.6
hsa-miR-17	101.8	120.9
